# Suppressing Lithium Polysulfide Shuttle in Li–S Batteries Using the AlPC_12_ Composite for Enhanced Stability and Performance

**DOI:** 10.1021/acsami.5c02942

**Published:** 2025-05-21

**Authors:** Ka Chun LI, Yaoqi WEI, Xuanming CHEN, Zeyuan DI, Chi Ho WONG, Leung Yuk Frank LAM, Xijun HU

**Affiliations:** a Department of Chemical and Biological Engineering, 58207 The Hong Kong University of Science and Technology, Kowloon 999077, Hong Kong, China; b Division of Science, Engineering and Health Studies, School of Professional Education and Executive Development, The Hong Kong Polytechnic University, Hong Kong, China

**Keywords:** lithium sulfur batteries, lithium polysulfide, aluminum alkoxide composite, high-performance Li−S batteries, lithium polysulfide adsorption

## Abstract

This study presents the aluminum phosphate composite (AlPC_12_) composite as a novel cathode material for lithium–sulfur (Li–S) batteries, addressing the polysulfide shuttle effect, a key challenge in Li–S battery performance. Synthesized via hydrolytic condensation and low-temperature calcination, the composite integrates aluminum alkoxide with phosphate to form a 3D structure that immobilizes lithium polysulfides (LiPS), enhancing battery efficiency and lifespan. Experimental analyses, including visible LiPS adsorption tests, and electrochemical measurements, demonstrate the superior performance of AlPC_12_ over traditional cathodes. Electrochemical tests show that AlPC_12_/S batteries exhibit exceptional discharge capacities and stability, outperforming titanium-based cathode and Super P cathode. At 0.5 C, the battery has an initial capacity of 837 mAh/g with a decay rate of 0.06% per cycle, and at 3 C, an initial capacity of 529 mAh/g with a decay rate of 0.08% per cycle. Increased sulfur loading does not affect LiPS control, with a 2.1 mg sulfur-loaded battery showing a decay rate of 0.03% over 750 cycles. Density functional theory (DFT) calculations confirm strong LiPS interactions, essential for efficient LiPS capture. This research promotes sustainability through a scalable, eco-friendly production process, minimizing environmental impact and advancing high-energy-density battery technologies.

## Graphical abstract

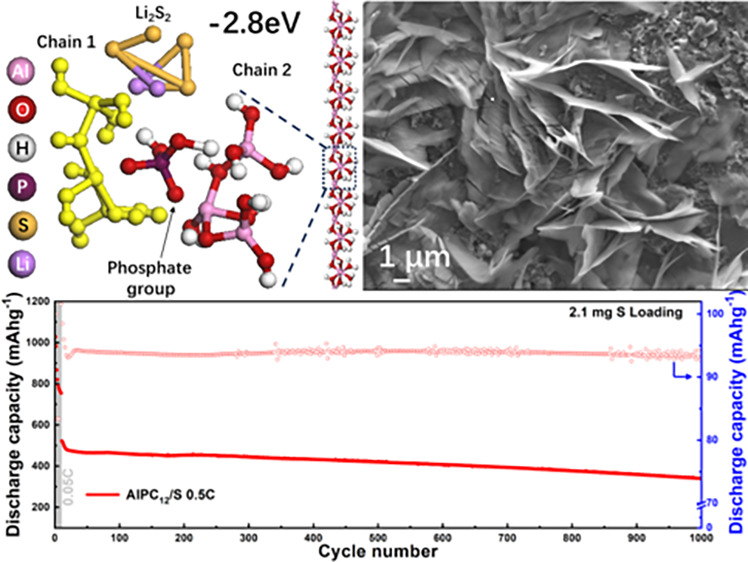

## Introduction

The surging demand for mobile devices, including mobile phones and electric vehicles, has necessitated the development of batteries with higher energy densities. In this landscape, lithium–sulfur (Li–S) batteries have emerged as a focal point of scientific interest, attributed to their exceptional theoretical specific capacity of 1675 mAh/g and energy density of 2600 Wh/kg.^1^ Such attributes position Li–S batteries as a promising solution to meet the increasing need for high-performance batteries. However, challenges such as the insulating nature of sulfur, cathode volume expansion postdischarge, and the ″polysulfide shuttle″ effect have been identified as significant impediments to their optimal performance. The ″polysulfide shuttle″ effect, characterized by the leaching of LiPS from sulfur into the electrolyte and their subsequent migration from the cathode to the anode, results in the formation of an insulating layer on the anode’s surface, thereby degrading battery performance.

To address the challenges associated with Li–S batteries, a variety of cathode materials have been explored. Carbon-based materials, such as nanostructured graphene,^2^ reduced graphene oxide,^3^ carbon nanotubes,^4^ and activated carbon^5^ are widely studied due to their excellent electrical conductivity and high porosity, which facilitate sulfur utilization and provide physical confinement for lithium polysulfides (LiPS). However, their nonpolar nature results in weak interactions with polar LiPS, leading to inadequate chemical anchoring and a rapid decline in capacity during long-term cycling.

To overcome this limitation, polar metal oxides, including SnO_2_,^6^ MnO_2_,^7^ Fe_2_O_3_,^8^ and TiO_2_,^9–13^ have been introduced as sulfur host materials. These oxides interact strongly with LiPS through Lewis acid base interactions, enhancing LiPS retention and suppressing the shuttle effect. Additionally, selenium-based materials have also demonstrated promising performance in Li–S batteries,^14,15^ although their applications are often constrained by complicated synthesis procedures and the need for hazardous chemicals or extreme conditions. More recently, aluminum-based materials have emerged as promising alternatives. For instance, Al_2_O_3_ ^16–18^ and aluminum-based metal–organic frameworks (Al-MOFs)^19^ have shown excellent chemical affinity toward LiPS, effectively mitigating the shuttle effect and improving electrochemical stability. Besides their strong polarity, aluminum-containing compounds benefit from being lightweight and earth-abundant, which are crucial attributes for developing high-energy-density and cost-effective batteries.

Phosphate compounds such as zirconium phosphate,^20,21^ iron phosphate,^22–24^ and aluminum phosphate^25^ have demonstrated an improved performance in Li–S battery. For example, Vu et al. used a sol–gel method to synthesize AlPO_4_ nanoparticles, which were employed as a cathode material for Li–S batteries^25^ and Chen et al. successfully synthesized a polymeric aluminophosphate binder to replace conventional PVDF in Li–S batteries.^26^ This affinity is crucial, as the significant polarity^22^ of phosphate compounds is instrumental in the chemical adsorption of LiPS, substantially reducing the shuttle effect, thereby extending battery life and enhancing efficiency. Furthermore, the electrochemical stability of phosphates, along with their compatibility with lithium,^21^ improves the battery’s performance and contributes to an increased energy density. Phosphates also offer environmental and economic benefits,^22^ meeting the demand for sustainable and cost-effective battery materials. These advantages establish phosphate compounds as a strategic choice for advancing Li–S battery technology, promising enhancements in performance, stability, and environmental sustainability.

Therefore, it is reasonable to further investigate aluminum phosphate-based materials as promising candidates for Li–S battery cathodes, given their combined advantages in polarity, stability, and structural tunability.

Except for the chemical compositions of the cathode materials, the role of three-dimensional (3D) structures in immobilizing LiPS has received comparatively less attention, despite extensive research on the chemical functionalities of metal oxides and phosphates. Inspired by the work of Xu et al., who developed a 3D sandwiched MoSe_2_ sulfur host,^27^ a novel three-dimensional aluminum alkoxide-phosphate composite (AlPC_12_) is reported in this study. In AlPC_12_, aluminum alkoxide chains are assembled into a cotton ball-like network. This 3D architecture is believed to contain internal voids and entangled channels that enable LiPS confinement not only through surface adsorption but also via physical entrapment within the structure. As a result, the shuttle effect can be effectively suppressed, leading to enhanced cycling stability.

AlPC_12_ is synthesized via a simple hydrolytic condensation process followed by low-temperature calcination, with tetrapropylammonium bromide serving as a structural template. This synthetic route avoids hazardous reagents and is amenable to scale-up, offering an economical and environmentally friendly approach. The resulting composite exhibits outstanding electrochemical performance as a sulfur host in Li–S batteries, extending their lifespan and promoting long-term operational stability. These advances contribute to overcoming key barriers in the commercialization of Li–S batteries by addressing challenges related to performance, cost, and sustainability.

## Experimental Section

### Synthesis of the AlPC_12_ Composite

The synthesis of the AlPC_12_ composite was carried out using phosphoric acid (H_3_PO_4_) and tetrapropylammonium bromide (TPAB) as the sources of phosphate and carbon, respectively. Initially, a solution containing 0.015 mol of aluminum isopropoxide was prepared in 18 mL of deionized (DDI) water to facilitate hydrolytic condensation. Subsequently, 0.0035 mol of H_3_PO_4_ was gradually added, resulting in a homogeneously turbid solution, designated as the aluminum phosphate precursor. In parallel, a carbon precursor solution was formulated by dissolving 0.003 mol of TPAB in 16 mL of DDI water. These two solutions were combined, followed by the addition of ammonia to attain a pH range of 8–9. The mixture was subjected to rigorous magnetic stirring for a duration extending between 24 and 48 h, leading to the formation of a white-colored precipitate. The precipitate was thoroughly rinsed with DDI water and dried at 100 °C overnight. The resultant dried powder was then calcined in a tube furnace under a nitrogen atmosphere at 350 °C for 2 h, with a heating rate of 2 °C/min.

### Synthesis of the AlPC_12_/S Composite

The AlPC_12_ and sulfur composite (AlPC_12_/S composite) was prepared using a melt-diffusion method. Specifically, the AlPC_12_ composite was first mixed well with sublimated sulfur at a weight ratio of 1:3 by ball milling for 4 h. The resulting mixture was then placed into a quartz boat and transferred into a tube furnace for heat treatment. The temperature was increased to 155 °C at a rate of 3 °C/min and maintained for 12 h under an argon environment. After the heat treatment, the sample was ground into powder using ball milling. The Super P/S composite was prepared with the same method for performance comparison.

### Thermogravimetric Analysis (TGA)

The sulfur content was determined using TGA. This analysis was conducted in a nitrogen atmosphere, with the temperature being gradually increased from 25 to 800 °C at a rate of 5 K/min.

### LiPS Adsorption Test

From the literature,^28,29^ a LiPS adsorption experiment was conducted to evaluate the adsorption efficiency of cathode materials, specifically using the AlPC_12_ composite. To prepare the 4 × 10^–3^ M Li_2_S_6_ solution, stoichiometric amounts of sublimated sulfur and lithium sulfide (Li_2_S) in a molar ratio of 5:1 were dissolved in a 1,2-dimethoxyethane (DME)/1,3-dioxolane (DOL) solution (v/v = 1:1) and magnetically stirred for 48 h, resulting in a deep-orange solution. Subsequently, 30 mg of the AlPC_12_ composite and 2.5 mL of the Li_2_S_6_ solution were added into a transparent container to allow adsorption to occur. The solution was shaken for the first 5 min and then stabilized inside a glovebox to prevent exposure to air.

### Electrochemical Measurements

The sulfur composites were mixed with conductive multiwalled carbon nanotubes (MWCNTs) and polyvinylidene fluoride (PVDF) in a weight ratio of 7:2:1 using ball milling. *N*-Methyl-2-pyrrolidone (NMP) solution was added to the mixture to form a toothpaste-like homogeneous slurry, which was then uniformly coated onto aluminum foil using the doctor blade technique. The coated foil was dried overnight at 60 °C in a vacuum oven and cut into 12 mm-diameter discs to serve as the cathode for the lithium–sulfur coin cells. The cells were assembled inside an argon-filled glovebox using Celgard 2325 separator and a lithium foil anode. The electrolyte was prepared by mixing DOL and DME in a volume ratio of 1:1, with 1 mol lithium bis­(trifluoromethanesulfonyl)­imide and 0.1 mol LiNO_3_. Adequate amounts of electrolyte, 20 μL (E/S ratio of ∼18 μL mg^–1^) for low-loaded cathodes and 40 μL (E/S ratio of ∼18 μL mg^–1^) for high-loaded cathodes, were used.^30^ The assembled batteries were subjected to galvanostatic analysis at different current densities between 1.7 and 2.8 V using a CT4008T cell test instrument (NEWARE, China), kept at a constant 25 °C temperature. Galvanostatic intermittent titration test (GITT) was performed at 0.05 C with 20 min of discharge/charge and 2 h of OCV rest.^31^ Cyclic voltammetry (CV) scanning of the assembled batteries was also conducted using an electrochemical workstation (CH Instruments, China) in the voltage range of 1.7–2.8 V.

### Visible Li–S Battery Test

A visible battery setup was created using a 20 mL glass bottle, stainless steel rods, and clips, as shown in Figure 1. The anode consisted of a lithium plate, while the cathode was a test cathode plate (AlPC_12_/S and Super P/S). The electrolyte was poured into the glass bottle to fully cover both the lithium and cathode plates. The assembly of the battery was carried out in an argon-filled glovebox, and it was sealed before being removed from the glovebox for testing. After the assembly, a discharge process was performed from 2.3 to 1.7 V. The battery was recorded on video to observe the changes.

**Figure 1:**
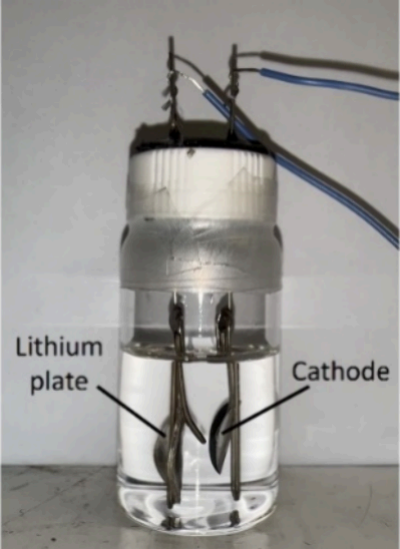
Visible Li–S battery setup.

### DFT Calculation

The calculations were executed using the CASTEP module. We employed the GGA-PBE functional^32^ and ultrasoft pseudopotential^33^ To investigate the formation of the AlPC_12_ composite and its subsequent interaction with Li_2_S_4_, we constructed a vacuum box measuring 20 Å × 20 Å × 15 Å.^34^ This design was chosen to minimize interactions between adjacent slabs. A basic linear hydrated alumina short-chain and dihydrogen phosphate formula were employed to represent the local structures after combining the relevant studies and practical conditions such as pH in our experiments.^35–37^ The basis set for the projector augmented plane-wave method was determined with a cutoff energy of 450 eV.^38^ A convergence threshold of 1 × 10^–6^ eV/atom was set for the self-consistent field (SCF) iterations.^39^ The adsorption energy (*E*_ads_) of Li_2_S_4_ on the AlPC_12_ surface is calculated using the formula *E*_ads_ = *E*_total_ – ELi_2_S_4_ – EAlPC_12_. Here, *E*_total_ is the total energy of the Li_2_S_4_ adsorbed on AlPC_12_, ELi_2_S_4_ is the energy of isolated Li_2_S_4_ in a vacuum, and EAlPC_12_ represents the energy of the optimized AlPC_12_ structure alone. A convergence test specific to the K-points has already been conducted, and a difference of less than 0.01 eV was observed.

## Results and Discussion

### Structure, Composition, and Chemical Interaction

The SEM was utilized to examine the morphology of the AlPC_12_ composite. Figure 2a presents a high-resolution SEM image of AlPC_12_, revealing its intricate structure. To offer a detailed view of the randomly distributed coniferous-like structure of the composite, an enlarged image is provided in Figure 2b. Some of these coniferous-like structures, approximately 50 nm in length, are observed to agglomerate, forming particulate composites as seen in Figure 2a. The morphology captured by SEM aligns with the expected product of aluminum isopropoxide (AIP) hydrolytic condensation, serving as evidence of the successful synthesis of the material. To further explore the elemental distribution within the AlPC_12_ composite, EDX mapping was employed. An aggregated composite is displayed in Figure 2c, while the distribution maps for P, O, and Al are shown in Figure 2d, Figure 2e, and Figure 2f, respectively. The elemental maps correlate with the shape of the composite, indicating a uniform distribution across the particulate structure. This uniformity in elemental distribution is crucial in understanding the composite’s composition and further validates the successful synthesis of AlPC_12_. The SEM image of the lithium anode after cycling in the AlPC_12_/S cell is shown in Figure S1. It reveals a relatively smooth and compact surface without apparent dendritic or mossy lithium deposits. No severe corrosion features or thick reaction layers were observed, indicating that the shuttle effect was effectively mitigated. These results suggest that the AlPC_12_ host successfully restricted the diffusion of LiPS to the anode side, thereby enhancing the cycling stability of the Li–S battery.

**Figure 2:**
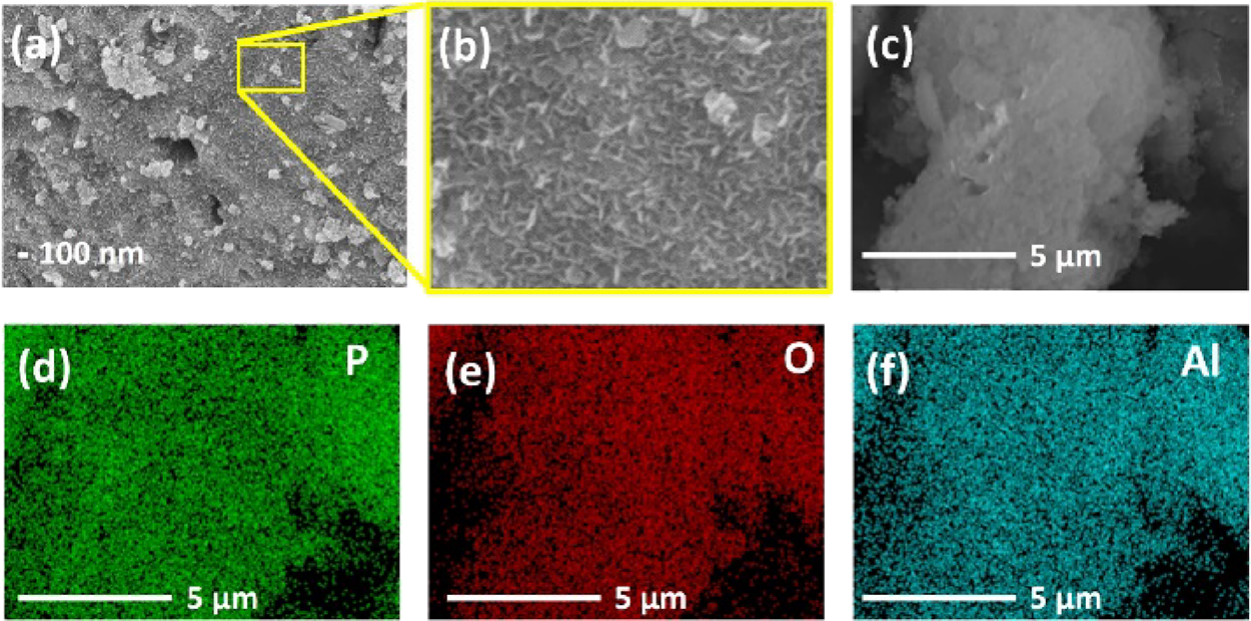
(a–c) SEM images of the AlPC_12_ composite and (d–f) its corresponding elemental mapping.

To further investigate the structural and compositional evolution of the AlPC_12_/S cathode, SEM and backscattered electron (BED-C) imaging were conducted before and after cycling. As shown in Figure 3a–d, the pristine AlPC_12_/S cathode (Figure 3a) exhibits a uniform and rough surface morphology, while its corresponding BED-C image (Figure 3b) reveals a homogeneous contrast across the surface, indicating an even distribution of constituent elements. After cycling, the AlPC_12_/S electrode (Figure 3c) develops a well-defined, layered, and crystalline structure, which may originate from the electrochemical activation of the composite. The BED-C image of the cycled electrode (Figure 3d) shows clear contrast variations, where the darker regions correspond to lighter elements such as C and O, while the brighter regions indicate heavier atoms like Al, P, and S. This elemental redistribution implies that AlPC_12_ undergoes a structural reconstruction that facilitates the chemical immobilization of LiPS. In addition, EDS mapping was performed on the cycled cathode to examine the elemental distribution of oxygen (e) and sulfur (f). It can be observed that the S signals significantly overlap with O signals, suggesting that sulfur species are uniformly adsorbed on the oxygen-containing surface of AlPC_12_. This result provides further evidence of the strong chemical interaction between the polar surface of AlPC_12_ and LiPS, contributing to effective shuttle suppression and enhanced cycling stability.

**Figure 3:**
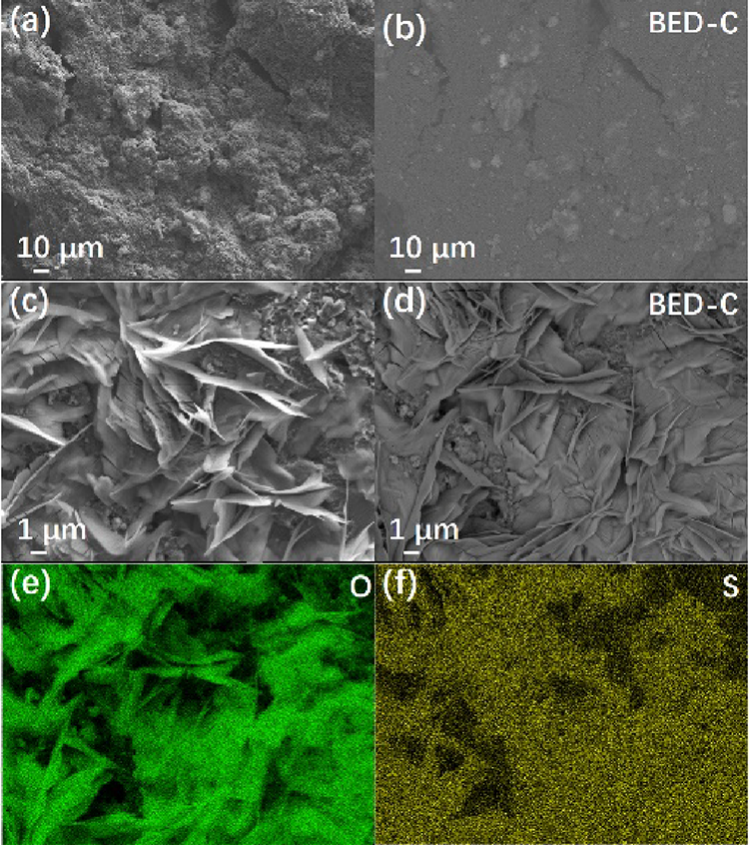
SEM images of AlPC_12_/S cathode before cycling (a) and its corresponding backscattered image (b). SEM image of the cathode after cycling (c) and the corresponding backscattered image (d). Elemental mappings of oxygen (e) and sulfur (f) for the cycled cathode.

The BET analysis revealed that the AlPC_12_ composite possesses a BET area of 66 m^2^/g and a pore volume of 0.44 cc/g. This porous structure enhances the interaction with sulfur, optimizing its utilization and offering superior adsorption sites for LiPS. The hysteresis loop of the composite is categorized as Type IV­(a) by IUPAC, as illustrated in Figure 4a. The BJH plot, shown in Figure 4b, highlights a predominant presence of pores in the 2–10 nm range, along with a minor peak between 10 and 100 nm, indicating the mesoporous structure of the composite. Additionally, the MP plot in Figure 4c further confirms the microporous nature, showing pore diameters ranging from 0.7 to 2 nm. The composite’s average pore size of 26.8 nm is optimal for sulfur storage and suitable for accommodating volume changes during Li_2_S_4_ and Li_2_S transitions. The control host, Super P, exhibits a BET surface area of 60 m^2^/g and a pore volume of 0.34 cc/g, with an adsorption isotherm featuring a Type IV­(a) hysteresis loop (Figure S2). These characteristics are comparable to those of the AlPC_12_ composite, establishing Super P as a suitable control host.

**Figure 4:**
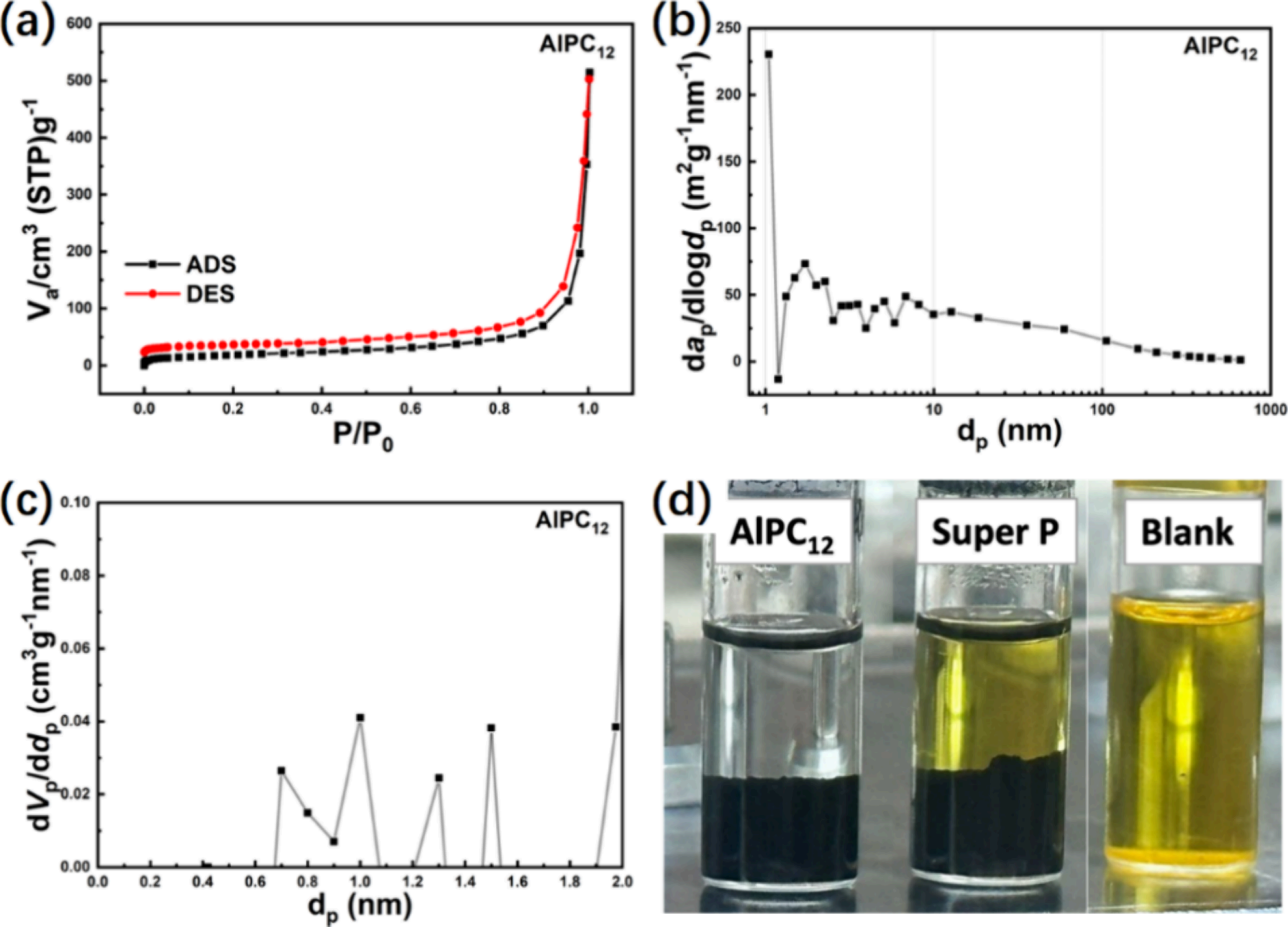
(a) BET isotherm, (b) BJH plot, (c) MP plot, and (d) visible adsorption test of AlPC_12_ composite and Super P.

TGA for the Super P/S composite and the TG curve are shown in Figure S3. As shown, the sulfur content in Super P/S is approximately 75 wt %, which is comparable with that of AlPC_12_/S (∼73.5 wt %). This confirms that both composites contain comparable sulfur loading, ensuring a fair performance comparison.

The synthesized AlPC_12_ exhibits an absence of distinct crystallization peaks. As shown in Figure S4, a comparative analysis between AlPC_12_ and the reference pattern of AlPO_4_ (#50-0303) indicates that the dominant peaks align at similar positions, suggesting that the synthesized material possesses a structural resemblance to AlPO_4_. The amorphous nature of AlPC_12_ is attributed to the disordered arrangement of alumina short chains of varying lengths. Additionally, the XRD pattern of AlPC_12_/S shows multiple crystallization peaks, with the main peak position aligning with the primary peak of sulfur (#08-0247). This alignment confirms that sulfur can be adsorbed onto AlPC_12_, indicating that AlPC_12_ serves as a stable sulfur host.

The FTIR spectrum of the sample labeled AIPC_12_ exhibits distinct characteristic peaks corresponding to various functional groups, shown in Figure 5b. The peak observed between 305 and 659 cm^–1^ corresponds predominantly to the O–P–O bending vibrations characteristic of phosphorus-based compounds.^40^ The peak at 648 cm^–1^, however, exhibits a notably lower intensity than other peaks associated with PO_4_ stretching and vibration, suggesting an influence from an additional component. Prior studies indicate that aluminum can disrupt the P–O–P network and instead form a more stable P–O–Al bond.^41^ Therefore, the FTIR spectrum provides critical insights into the chemical composition of the AlPC_12_ sample, supporting the presence of an Al–O–P linkage between aluminum and phosphorus.

**Figure 5:**
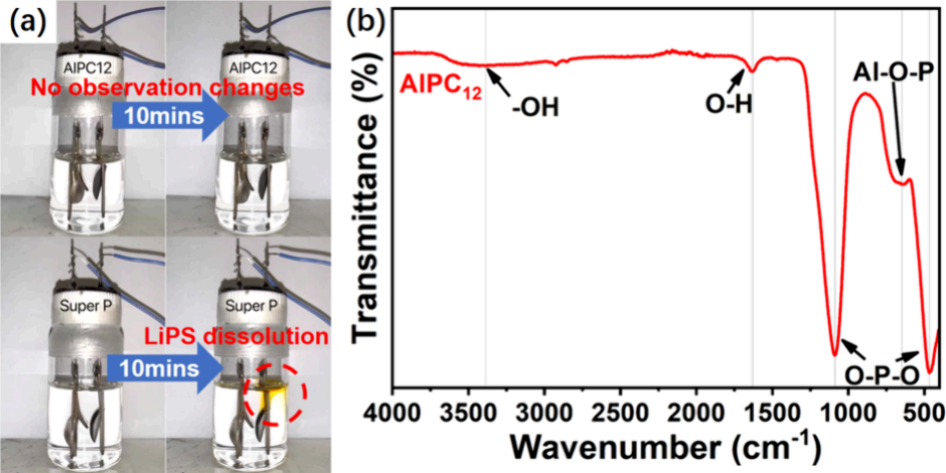
(a) Visible Li–S battery test. (b) FTIR spectrum of AlPC_12_.

Furthermore, the spectrum displays a sharp peak at 1087 cm^–1^, attributed to the asymmetric stretching of PO_4_, and a peak around 466 cm^–1^.^42^ The peak observed between 305 and 659 cm^–1^ typically corresponds to the O–P–O bending vibrations characteristic of phosphorus compounds.^40^ Notably, the peak intensity at 648 cm^–1^ is weaker than that of the other peaks associated with the stretching and vibration of PO4, implying that this peak may be influenced by another component. Research has shown that aluminum can disrupt the P–O–P network and form a more stable P–O–Al bond.^41^ The FTIR spectrum thus provides valuable insights into the complex chemical composition of the AlPC_12_ sample, confirming that aluminum and phosphorus are connected through an Al–O–P structure.

To evaluate the LiPS adsorption capability of AlPC_12_, an adsorption test was conducted inside an argon-filled glovebox. AlPC_12_ and Super P were added to the LiPS solution to assess adsorption and observe any resultant color change in the solution. The strong affinity of AlPC_12_ for LiPS was demonstrated, as illustrated in Figure 4d. The LiPS solution, initially orange in color, became colorless upon interaction with AlPC_12_. Conversely, no change was observed in the solution when Super P was introduced. This comparison highlights the superior LiPS adsorption capacity of AlPC_12_ relative to Super P. Similar findings were observed during the visible battery test, as depicted in Figure 5a and further detailed in the video available in the supplementary documents. In the setup utilizing AlPC_12_/S, no LiPS dissolution was observed throughout the whole discharge process, resulting in the electrolyte remaining clear and transparent. Conversely, the setup containing Super P/S exhibited noticeable LiPS dissolution from the cathode plate, characterized by the release of a deep-yellow substance indicative of LiPS.

The XPS spectrum has provided insight into the chemical interactions within the AlPC_12_ composite and AlPC_12_/S composite from the setup after adsorption test, as detailed in Figure 6. The comparison of the two curves in Figure 6a reveals the presence of sulfur’s 2p peak at 164.3 eV and 2s peak at 226.2 eV in the AlPC_12_/S spectrum, indicating that sulfur has been successfully loaded onto AlPC_12_. The presence of a peak at 74.4 eV in the Al 2p spectrum suggests the formation of aluminum alkoxide (O–Al–O), likely arising from AlP.^43^ This peak is characterized by its nearly symmetrical shape and a width at half-maximum of 1.95 eV, indicative of the specific aluminum environment in the composite. The observed binding energy for O–Al–O is lower than that typically associated with Al_2_O_3_, which may be ascribed to the coordination involving hydroxyl, phosphate, and aluminum groups.^44^ Moreover, the shift to higher binding energies compared to Al­(OH)_3_ could be indicative of a reduced rate of AlP hydrolysis condensation.^45^ The spectral signatures of Al 2p in Al_2_O_3_, AlOOH, and Al­(OH)_3_ are closely aligned, presenting a challenge in differentiation.^46^

**Figure 6:**
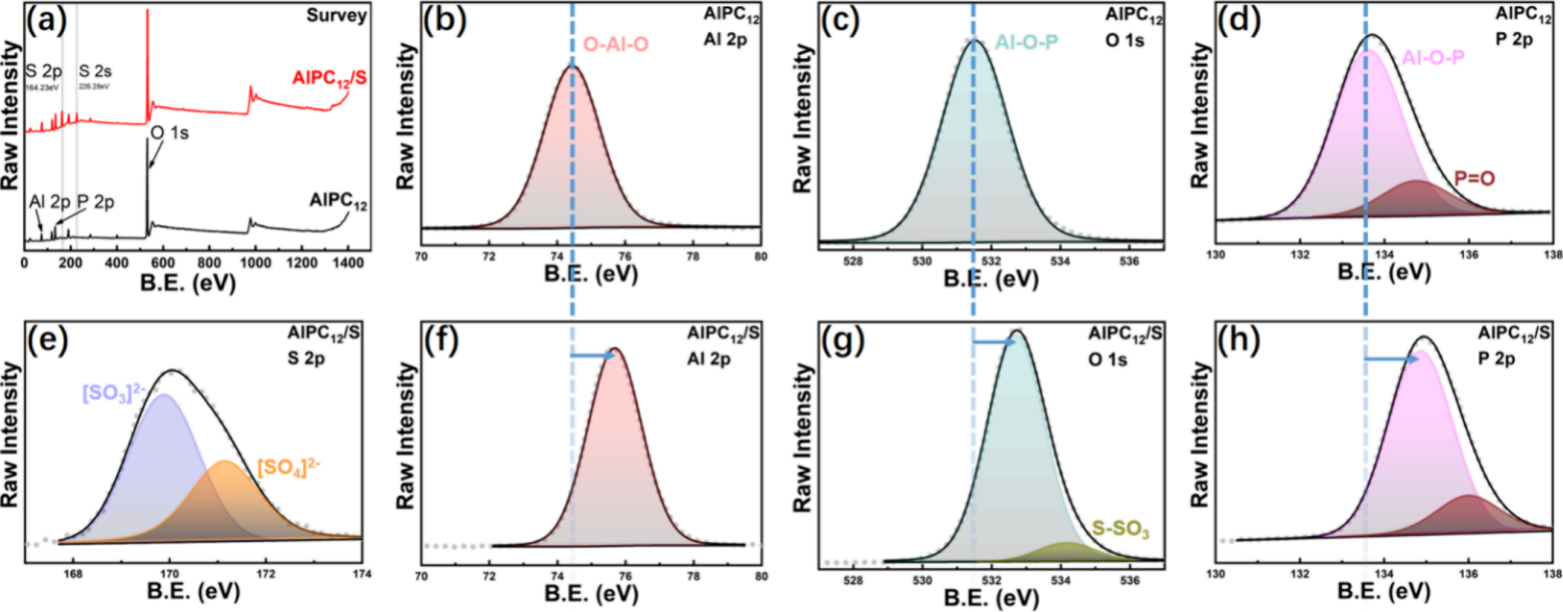
(a) XPS spectra of AlPC_12_ and AlPC_12_/S composites. (b–h) High-resolution XPS spectra of AlPC_12_ and AlPC_12_/S for different elements.

The O 1s spectrum exhibits a peak at 531.5 eV, corresponding to an Al–O bond with an acidic character, with a noted binding energy difference of 456.9 ± 0.1 eV.^47^ Additionally, a slight shift of 0.1 eV in this peak, associated with the interaction with the phosphate group, distinguishes it from those of hydroxyl groups.^48^ The presence of the Al–O–P bond is further corroborated by the peaks at 133.6 and 134.7 eV in the P 2p spectrum, which are linked to the enhanced intensity of the O 1s peak and display a lower binding energy than traditional aluminum phosphate.^49^ The separation of these two peaks should be due to the superimposed phenomenon from P atoms.^50^ A little amount of the partial reacted H_3_PO_4_ precursor held the P=O double bond and made those P atoms with a higher oxidation state, which can explain the small peak appearing at 134.7 eV, and consistent with the reference about AlPO_4_.^51^ These spectral features collectively provide evidence for the successful synthesis of AlPC_12_, highlighting the presence of an Al–O–P bond consistent with DFT calculation models.

For the AlPC_12_/S composite, all peaks observed in the AlPC_12_ composite are retained, and additional peaks related to sulfur are identified. Specifically, in the O 1s spectrum, an additional peak at 532.5 eV is observed, corresponding to the oxygen environment in sulfate, including thiosulfate and polythionate.^52^ This suggests the adsorption of LiPS species, promoting the interaction between sulfur from LiPS and oxygen atoms in the phosphate group. In the S 2p spectrum, peaks at 168.5 and 169.5 eV correspond to SO_3_^2–^ and SO_4_^2–^ groups,^53^ which are consistent with the O 1s spectrum, indicating the interaction between LiPS and the phosphate group. This interaction explains the superior LiPS adsorption performance of AlPC_12_.

In neutral conditions, AlP undergoes a hydrolysis process that leads to the formation of aluminum alkoxide chains, where aluminum exhibits a coordination number of six.^54^ Figure 7a illustrates the one-dimensional (1D) polymer chain of the aluminum alkoxide, consisting of several linked monomers. This structure, as computed by the CASTEP module, represents the most stable energy conformation. This configuration results in aluminum atoms pushing electrons toward the connected oxygen atoms, thereby creating areas of electronegativity on the surface of the chains. Further hydrolysis yields aluminum alcoholates, characterized by Al and OCH­(CH_3_)_2_ groups.^43^ Prior to the complete hydrolysis of AlP, H_3_PO_4_ is introduced to reduce the pH of the reaction mixture. Within a pH range of 2 to 7, there is a strong attraction between Al species and phosphate groups due to hydrogen bonding.^55^ This interaction significantly facilitates the formation of bonds between phosphate anions and aluminum cations. Supporting this observation, a peak at 637 cm^–1^ identified in the FTIR results corresponds to Al–O–P stretching vibrations. This peak provides further evidence of the chemical interactions and bond formation within the composite, aligning with the proposed mechanism between aluminum and phosphate groups. In the formation of the AlPC_12_ composite, aluminum alkoxide chains are interconnected through phosphate ions, serving as junction points, as depicted in the schematic diagram presented in Figure 7b. This model reveals that the chains align at an angle of 46.5° to achieve the lowest energy configuration. Given the multitude of potential connection points along each polymer chain, the resulting overall structure of the composite is predicted to be amorphous. This amorphous nature accounts for the absence of distinct peaks in the XRD analysis, aligning with the expected structural characteristics of such a composite.

**Figure 7:**
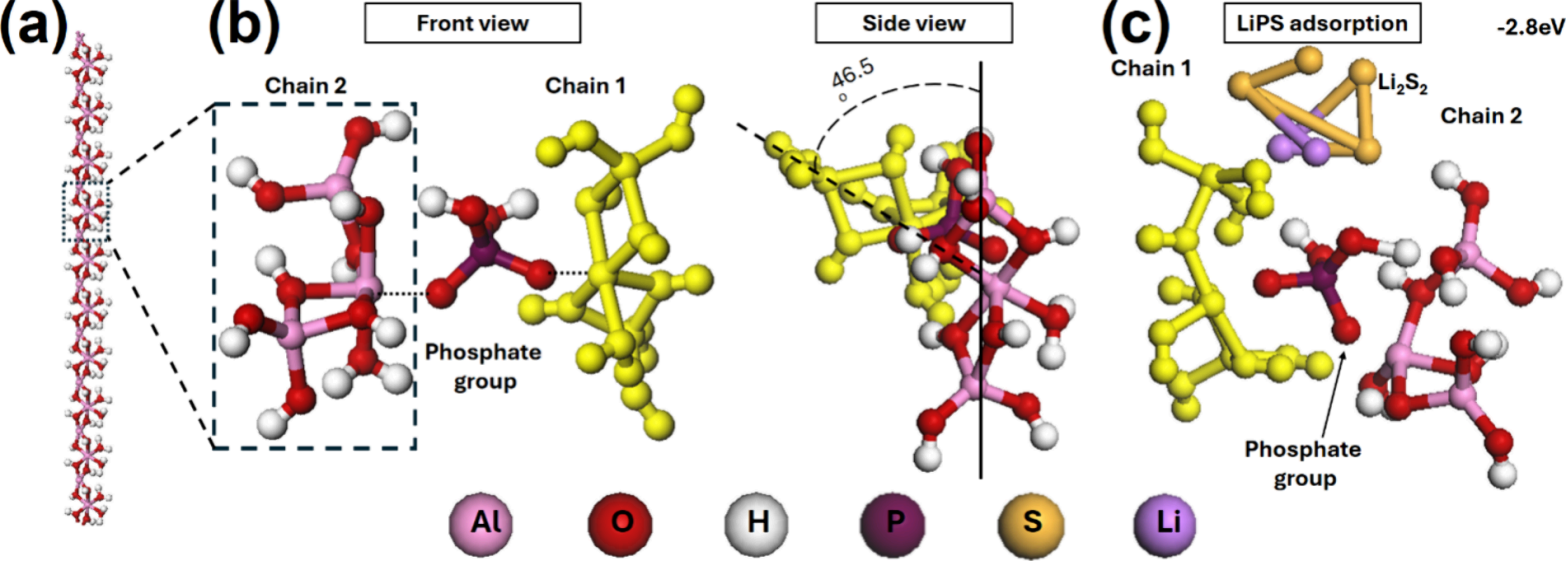
Schematic diagram of (a) 1D aluminum alkoxide chain, (b) conjunction between two aluminum alkoxide chains, and (c) adsorption of LiPS of the AlPC_12_ composite.

The AlPC_12_ model was developed based on structural transformations identified in previous studies on the hydrolysis of AlP, alongside FTIR data and XPS results confirming the presence of Al–O–P structures.^37,56^ In this system, interactions between H_2_PO_4_^–^ ions and the polymeric chains formed posthydrolysis of AlP facilitate chain linkages. This phenomenon aligns with the bidentate binuclear interaction noted by Luengo et al. in earlier literature.^37^ The model also demonstrates that AlPC_12_ exhibits a strong affinity for lithium–sulfur compounds, highlighting its potential as a candidate material for lithium–sulfur battery applications. Although XRD analysis confirms AlPC_12_’s amorphous nature, FTIR spectroscopy, which primarily assesses surface characteristics, reveals the presence of Al–O–P groups on the AlPC_12_ surface. The FTIR peak associated with Al–O–P stretching vibrations further substantiates the presence of Al–O–P linkages.^57^ Additionally, the material’s BET surface area of 66.3 m^2^/g and pore size of 26.8 nm indicate a substantial specific surface area and suitably dimensioned pore channels, facilitating the ingress and egress of lithium polysulfide. These features provide optimal conditions for polysulfide interaction, thereby promoting efficient lithium–sulfur exchange during battery charge and discharge cycles. This reinforces the feasibility of using AlPC_12_ in lithium–sulfur batteries.

To further investigate the adsorption behavior of LiPS on the AlPC_12_ composite, density functional theory (DFT) simulations were conducted for four representative LiPS species: Li_2_S_2_, Li_2_S_4_, Li_2_S_6_, and Li_2_S_8_. The total energy of each system was calculated to evaluate the adsorption strength. The findings show that all adsorption processes are exothermic, indicating favorable interactions between the AlPC_12_ composite and LiPS molecules. Among the four species, Li_2_S_2_ exhibits the highest adsorption energy at −2.98 eV, followed by Li_2_S_4_ (−2.84 eV), Li_2_S_6_ (−2.37 eV), and Li_2_S_8_ (−2.01 eV). This trend suggests that the AlPC_12_ composite interacts more strongly with short-chain polysulfides, while still maintaining sufficient affinity toward long-chain species such as Li_2_S_8_. The decreasing adsorption energy with increasing chain length also implies a dynamic interaction, where long-chain LiPS are more easily released during charging and effectively trapped during discharge. This behavior supports the composite’s suitability for lithium–sulfur battery applications by helping to suppress the shuttle effect. As illustrated in the schematic diagram (Figure 7c), the adsorption sites for LiPS are strategically located at the junctions formed by multiple phosphate ions connecting numerous alkoxide chains. This unique structural arrangement creates a three-dimensional network of adsorption sites throughout the AlPC_12_ bulk, enabling LiPS molecules to be confined within the composite. Such confinement prevents their dissolution into the electrolyte, effectively mitigating the shuttle effect. Consequently, the strong and stable adsorption behavior of AlPC_12_ toward LiPS plays a critical role in improving the electrochemical stability, cycling performance, and overall efficiency of Li–S batteries.

### Electrochemical Performance

Electrochemical analyses were conducted to evaluate the performance of the AlPC_12_ as a cathode material for lithium–sulfur batteries.

Figure 8a and Figure 8b present the CV curves of Li–S batteries employing AlPC_12_ and Super P as sulfur host materials, respectively. Each curve exhibits the characteristic four redox peaks associated with lithium–sulfur electrochemistry, comprising two cathodic and two anodic peaks. During the cathodic scan, the peak observed at approximately 2.3 V corresponds to the reduction of elemental sulfur to higher-order LiPS, while the peak near 2.0 V reflects the further reduction to lower-order LiPS, culminating in the formation of Li_2_S_2_/Li_2_S. In the anodic scan, the oxidation peak around 2.3 V is attributed to the transformation of lower-order LiPS back to higher-order LiPS, whereas the peak near 2.45 V denotes the subsequent oxidation to elemental sulfur.

**Figure 8:**
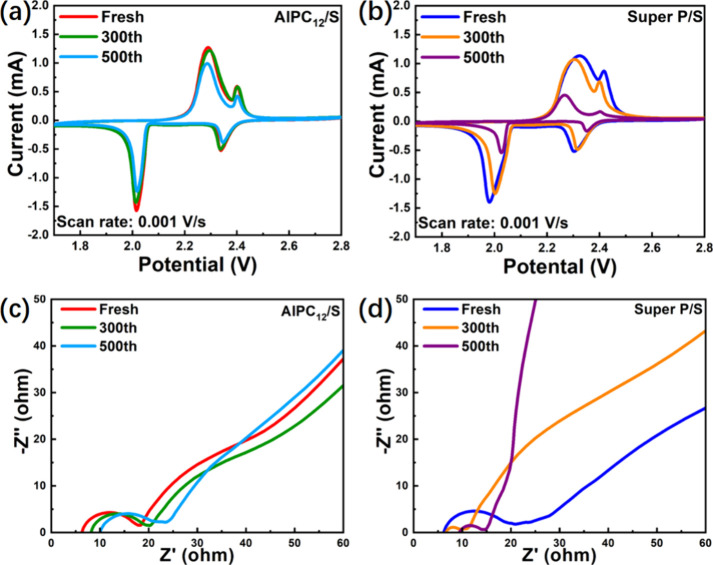
CV curves of (a) AlPC_12_/S battery and (b) Super P/S battery. Nyquist plots of (c) AlPC_12_/S battery and (d) Super P/S battery.

A comparison of the first-cycle CV curves of the AlPC_12_/S and Super P/S electrodes is provided in Figure S5. The first-cycle CV curves of the AlPC_12_/S and Super P electrodes exhibit similar reduction peak currents, with no distinct advantage observed for Super P in the negative current region. Notably, the AlPC_12_/S electrode shows a sharper oxidation peak and a relatively narrow peak separation, indicating lower polarization and improved reaction kinetics. These findings suggest that AlPC_12_/S may provide enhanced reversibility and more effective sulfur utilization.

In Figure 8a, the CV profiles of the AlPC_12_/S battery exhibit significant overlap between the initial and 300th cycles, with only marginal decreases in intensity observed after 500 cycles. Conversely, the Super P/S battery (Figure 8b) displays a pronounced attenuation in peak intensity, with values nearly halved after 500 cycles. Notably, the peak positions of the AlPC_12_/S electrode remain stable throughout cycling, indicating enhanced electrochemical reversibility. In contrast, the Super P/S electrode reveals evident peak shifts, further confirming its inferior cycling stability. The sharper and narrower peaks observed for the AlPC_12_/S battery also suggest improved electron transfer kinetics, likely facilitated by the 3D alkoxide framework. This structure promotes effective LiPS capture and confines active species near the sulfur host surface, thereby reducing electron transfer distances and mitigating capacity fade over extended cycling.

Electrochemical impedance spectroscopy (EIS) was conducted to investigate the electrochemical kinetics of both AlPC_12_/S and Super P/S batteries at various cycling stages, as shown in Figure 8c,d. The Nyquist plots of both systems display a semicircle in the high-frequency region, representing the charge transfer resistance (*R*_ct_), and a sloped line in the low-frequency region, corresponding to the Warburg impedance (*Z*_w_), which reflects lithium-ion diffusion.^58^ Notably, none of the plots exhibit the second depressed semicircle typically associated with Li_2_S_2_/Li_2_S accumulation, suggesting effective catalytic conversion and suppression of insulating species,^59^ particularly for the AlPC_12_ electrode.

In the fresh state, both AlPC_12_/S and Super P/S cells exhibit similar *R*_ct_ values, indicating comparable initial interfacial charge transfer kinetics. However, by the 300th cycle, the *R*_ct_ of the Super P/S cell significantly decreases, while the slope of *Z*_w_ becomes steeper, implying that although electronic conductivity temporarily improves due to the deposition of conductive Li_2_S across the Super P network, lithium-ion diffusion becomes increasingly hindered, likely due to uncontrolled LiPS redistribution and deposition outside the electrode framework. In contrast, the AlPC_12_/S cell at the 300th cycle retains a relatively consistent *R*_ct_ and *Z*_w_ slope, demonstrating stable electrode–electrolyte interfaces and effective confinement of sulfur species within the cathode. By the 500th cycle, the Super P/S cell exhibits a further decrease in *R*_ct_, but the continued steepening of the Warburg region highlights a growing limitation in ion transport, consistent with severe LiPS shuttle and structural degradation. On the other hand, the AlPC_12_/S cell shows only a slight increase in *R*_ct_, likely due to gradual accumulation of insulating discharge products (e.g., Li_2_S) on the catalytic surface. Nevertheless, its *Z*_w_ slope remains moderate, indicating preserved ion diffusion pathways. This overall stability suggests that the AlPC_12_ composite effectively maintains structural and electrochemical integrity over long-term cycling, offering superior performance in suppressing LiPS shuttle and enhancing electrode durability.

Figure S6 demonstrates that the proposed equivalent circuit achieves an effective fit, indicated by values of *x*_2_/*Z* < 0.1. The characteristic kinetic and transport resistances are connected to the measured EIS spectra by defining *R*_1_, *R*_2_, *R*_3_, and *W*_i_ as the resistances associated with the electrolyte, charge transfer, Li_2_S film, and Warburg impedance, respectively. It should be noted that the formation of an anode SEI layer or a passivation layer, influenced by the presence of LiNO_3_ in the electrolyte, may also contribute to the overall impedance of the Li–S cell. Previous studies have indicated that the high-frequency semicircle observed in EIS spectra may correspond to these anode resistances, potentially being predominant during the first discharge cycle. Nevertheless, it is typically the cathode that predominantly governs the impedance in Li–S batteries.

The galvanostatic intermittent titration test (GITT) studies of AlPC_12_/S and Super P/S with similar sulfur loadings, shown in Figure S7, elucidate the kinetic and thermodynamic advantages of AlPC_12_ over Super P. Both GITT profiles exhibit characteristic discharge–charge curves of Li–S batteries. However, the AlPC_12_/S electrode demonstrates more stable and higher plateau capacities with minimal polarization, indicating superior reaction kinetics. Furthermore, the deviation between the practical voltages at constant current pulse and the equilibrium voltages at steady state, as a function of the state of discharge and charge, reflects the chemical diffusion coefficient of the electrode during the relaxation process. The voltage of AlPC_12_/S generally relaxes more rapidly to equilibrium with significantly smaller voltage deviation, particularly during the discharge process.^31^ This suggests a high diffusion coefficient and a high concentration of polysulfides strongly bound to the AlPC_12_ electrode.

Figure 9a depicts the galvanostatic charge and discharge profiles of the AlPC_12_/S battery across various cycle numbers. The discharge curves reveal two distinct plateaus, indicative of a two-step reaction in the sulfur cathode. The initial plateau at 2.36 V corresponds to the redox reaction where sulfur transitions into long-chain lithium LiPS. The following plateau at 2.10 V represents the conversion of these long-chain LiPS into shorter-chain LiPS.^10^ These profiles are crucial for understanding the polarization potential, which is the voltage difference between charge and discharge. A smaller polarization potential indicates diminished internal resistance and improved reaction kinetics, while a larger value suggests the opposite. For the AlPC_12_/S batteries, polarization values during the 100th, 300th, and 500th cycles at 2 C were 0.227, 0.225, and 0.232 V, respectively, in contrast to the Super P/S battery, which registered 0.377, 0.415, and 0.395 V (Figure S8), Figure S9 presents the first-cycle galvanostatic charge–discharge profiles of Li–S batteries using AlPC_12_ and Super P as sulfur hosts at 0.05 C. Both profiles exhibit the typical two-plateau discharge characteristics of Li–S systems. The initial discharge capacity of the Super P/S battery is slightly higher (∼1200 mAh/g) than that of AlPC_12_/S (∼950 mAh/g), which is mainly due to the superior electrical conductivity of Super P that facilitates rapid electron transfer and sulfur activation in the early stage. However, this early advantage is temporary. The more gradual voltage plateaus and smoother charge profile of AlPC_12_/S suggest a more stable redox environment and reduced LiPS shuttle effect. This can be attributed to the strong polysulfide affinity and trapping capability of the AlPC_12_ composite, which confines active species within the cathode region and enhances sulfur utilization. Therefore, while Super P exhibits higher capacity initially, the AlPC_12_/S electrode is expected to outperform in long-term cycling due to its superior shuttle suppression and structural stability.

**Figure 9:**
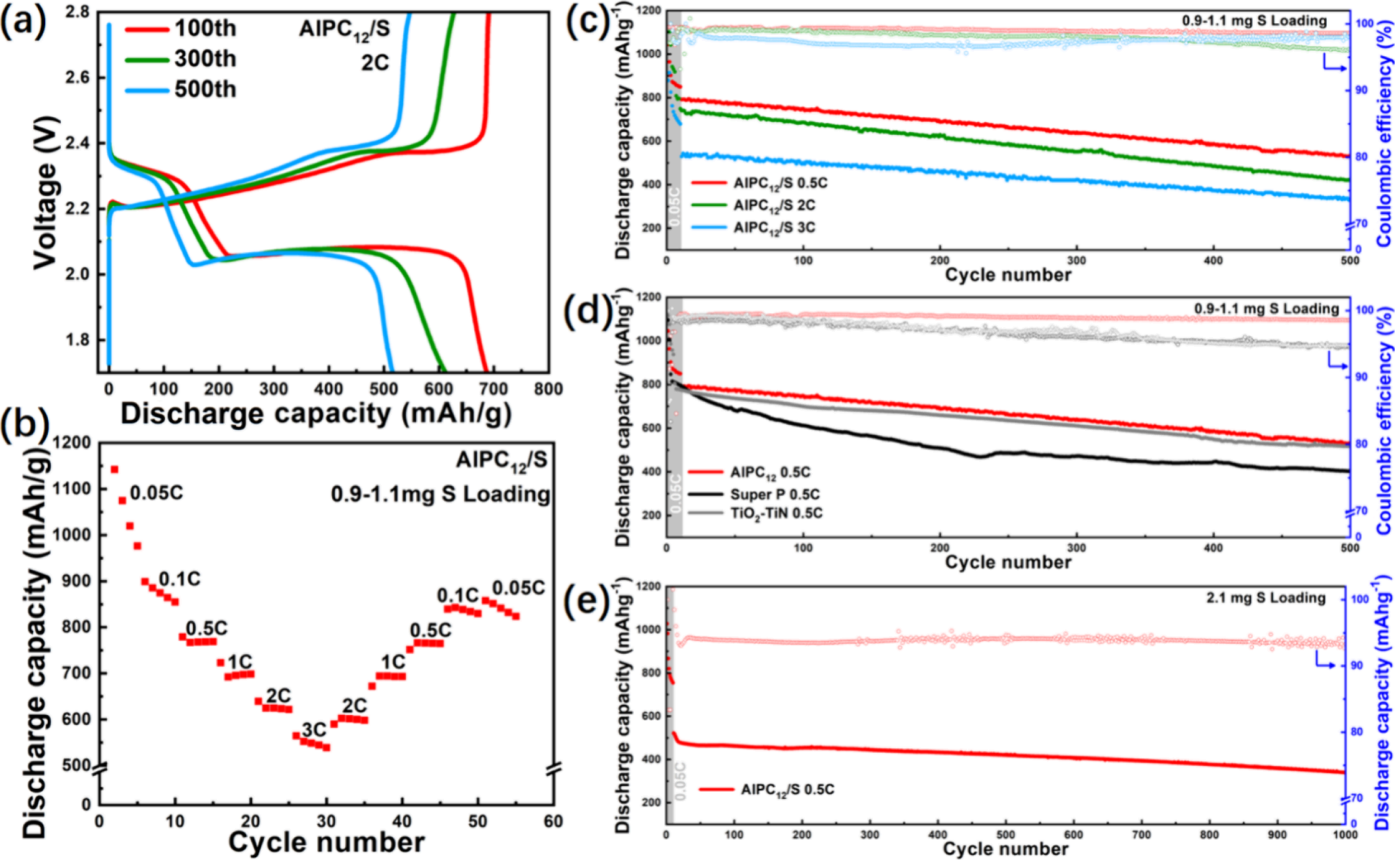
Electrochemical performance of lithium–sulfur batteries. (a) Galvanostatic charge and discharge profiles of the AlPC_12_/S battery at different cycle numbers in 2C. (b) Rate performance of AlPC_12_/S battery. (c) Cyclic charge–discharge performance test of the AlPC_12_/S battery in 0.5, 2, and 3 C. (d) Cyclic charge–discharge performance test of the AlPC_12_/S battery, TiO_2_–TiN/S, and Super P/S battery in 0.5 C. (e) Cyclic charge–discharge performance test of AlPC_12_/S batteries in 0.5 C with 2.1 and 4.3 mg S loading.

Figure 9b shows the rate capability of the AlPC_12_/S batteries at different current densities. The AlPC_12_/S battery displays discharge capacities of 1302, 899, 779, 723, 639, and 564 mAh/g at current densities of 0.05, 0.1, 0.5, 1, 2, and 3 C, respectively. When returned to their initial current densities in subsequent cycles, the battery maintains rate capabilities of 86.4, 97.8, 91.5, and 93.9% at 0.05, 0.1, 0.5, and 1 C, respectively. The superior rate capability of the AlPC_12_/S battery can be attributed to the effective interaction between the composite and LiPS, fostering efficient electrochemical reactions throughout the system. The rate performance of the Super P/S cathode is presented in Figure S10 for direct comparison. As shown, the Super P electrode delivers a higher initial discharge capacity, indicated by a longer plateau region between approximately 2.3 and 2.0 V. In contrast, the AlPC_12_/S electrode exhibits a slightly lower capacity but maintains a more stable voltage plateau, which could reflect differences in sulfur utilization and reaction kinetics. These results suggest that Super P may offer higher sulfur utilization during the first cycle, whereas AlPC_12_/S may provide a more stable electrochemical environment that could benefit longer-term cycling, and it is confirmed by the cyclic performance test results.

Figure 9c illustrates the cyclic performance of the AlPC_12_/S batteries tested at 0.5, 2, and 3 C rates. Initially, each battery underwent 10 conditioning cycles at a rate of 0.05 C. This conditioning process, involving slow charging and discharging, is critical for ensuring proper activation of the electrodes and uniform distribution of the electrolyte throughout the cell. The significant drop in discharge capacity observed during these conditioning cycles is attributed to the discharge of free sulfur, which is not bonded with the sulfur host, converting into LiPS and subsequently diminishing battery performance. This phenomenon is commonly encountered in Li–S battery testing. At a 0.5 C rate, the initial capacity of the AlPC_12_/S battery is 791.2 mAh/g, which decreases to 530.6 mAh/g after 500 cycles, resulting in an average decay rate of 0.06% per cycle. When operated at a 2 C rate, the battery’s initial capacity of 740 mAh/g reduces to 420 mAh/g over 500 cycles, corresponding to a decay rate of 0.09% per cycle. Similarly, at a 3 C rate, the battery begins with a capacity of 529 mAh/g, declining to 330 mAh/g over 500 cycles, with a relatively low decay rate of 0.08% per cycle. Notably, the battery at 0.5 C consistently achieves a Coulombic efficiency above 98%, whereas the 2 and 3 C batteries maintain an efficiency above 96%, underscoring the composite’s excellent control over LiPS.

Figure 9d compares the performance of AlPC_12_, Super P carbon, and a TiO_2_–TiN composite,^60^ which was synthesized and previously reported by our team, as sulfur hosts in Li–S batteries at a 0.5 C rate. The AlPC_12_/S battery exhibits the highest discharge capacity, significantly surpassing the Super P/S battery and marginally exceeding the performance of the TiO_2_–TiN composite. Specifically, the Super P/S battery shows a decrease in discharge capacity from 809 to 402 mAh/g over 500 cycles at 0.5 C, corresponding to a decay rate of 0.1% per cycle. This decay rate is substantially higher than that of the AlPC_12_/S battery, which is 0.06% per cycle. In comparison, the TiO_2_–TiN composite battery begins with a capacity of 774 and ends at 517 mAh/g after 500 cycles, with an average decay rate of 0.07% per cycle.^60^ Thus, the AlPC_12_/S battery demonstrates superior performance, establishing it as the most effective among the tested materials, slightly better than the TiO_2_–TiN composite and considerably more effective than Super P.

The cyclic performance of the AlPC_12_/S battery with sulfur loadings of 2.1 and 4.3 mg is presented in Figure 9e. For the battery with a sulfur loading of 2.1 mg, an initial discharge capacity of 521.5 mAh/g was observed, which decreased to 385.6 mAh/g after 750 cycles. The average decay rate was determined to be 0.03% per cycle. Similarly, the battery with a sulfur loading of 4.3 mg exhibited an initial discharge capacity of 548.7 mAh/g, which decreased to 25.2 mAh/g after 750 cycles, corresponding to an average decay rate of 0.12% per cycle. Notably, this decay rate is significantly lower than the values reported in Table 2 listed below. These results highlight the superior performance of the AlPC_12_ composite in suppressing LiPS shuttling, thereby ensuring enhanced long-term cycling stability of the battery.

LiNO_3_ has been widely reported to effectively suppress the polysulfide shuttle in Li–S batteries. To further evaluate the inherent capability of AlPC_12_ in mitigating the shuttle effect, a cycling performance test was conducted using AlPC_12_/S cathodes with an electrolyte without the addition of LiNO_3_ at a rate of 2 C. As shown in Figure S11, only a slight reduction in discharge capacity was observed compared to the system containing LiNO_3_, with a difference of less than 100 mAh/g over 200 cycles. This negligible decline suggests that effective confinement of LiPS was achieved by the AlPC_12_ host alone. The polar surface and three-dimensional alkoxide framework of AlPC_12_ are able to have contributed significantly to LiPS adsorption, thereby minimizing shuttle-related losses even in the absence of LiNO_3_.

To investigate the self-discharge behavior, the open-circuit potential (OCP) of the Li–S cells using AlPC_12_ and Super P as sulfur hosts was monitored over time, as shown in Figure S12. The cell assembled with Super P exhibited a continuous decline in OCP, indicating a significant self-discharge phenomenon due to the dissolution and diffusion of LiPS. In contrast, the AlPC_12_-based cell maintained a nearly constant OCP throughout the entire duration (∼5000 min), suggesting a much lower rate of self-discharge. This stable potential profile implies that LiPS were effectively immobilized within the cathode region, likely attributed to the strong adsorption capacity and polar interactions provided by the AlPC_12_ composite. These findings further confirm the ability of AlPC_12_ to suppress LiPS shuttle and enhance the electrochemical stability of the Li–S battery.

The catalytic performance of AlPC_12_/S and Super/S batteries in electrochemical reactions was further explored by determining the diffusion coefficient of lithium ions. This was accomplished by conducting a series of CV tests across scan rates between 0.1 and 0.4 mV·s^–1^ for both fresh and 500 cycled samples. The calculation of the Li^+^ diffusion coefficient was based on the Randles–Sevick equation (eq 1), which connects the peak current with the scan rate:

$$i_{p}=2.69\times 10^{5}n^{3/2}AD^{1/2}Cv^{1/2}$$ 1

In this equation, *i*_p_ represents the peak current, *n* denotes the number of electrons per reaction, *A* is the active electrode surface area, *D* is the diffusion coefficient of lithium ions, and *C* stands for the concentration of Li^+^ ions in the electrolyte. A linear correlation was observed between *i*_p_ and the square root of the scan rate *v* ^1/2^ for cathodic peaks around ∼2.3 V (A peaks) and ∼2.1 V (B peaks), as well as for anodic peaks near 2.3 V (C peaks) for both fresh and 500-cycled batteries. The corresponding lithium-ion diffusion coefficients were organized in Table 1. It presents the lithium-ion diffusion coefficients for two types of lithium–sulfur batteries, Super P and AlPC_12_/S, both in their fresh states and after 500 cycles. The results show that the diffusion coefficients for both batteries decrease after 500 cycles, indicating a reduction in lithium-ion mobility over time. However, when comparing the fresh batteries, AlPC_12_/S exhibits higher diffusion coefficient values than Super P across all three measurements (denoted as *D*_Li_^A^, *D*_Li_^B^, and *D*_Li_^C^). Since lithium-ion diffusion directly influences the kinetics of electrochemical redox reactions, a higher diffusion coefficient suggests enhanced ion transport and more favorable catalytic behavior within the electrode. Even after 500 cycles, AlPC_12_/S maintains higher diffusion coefficients than Super P, with a smaller percentage drop in values compared to Super P. These observations are consistent with the results from the GITT test, which also suggests that AlPC_12_/S demonstrates better long-term electrochemical stability and lithium-ion diffusion efficiency compared to Super P.

**Table 1:** Comparison of Lithium Diffusion Coefficients for Super P and AlPC_12_/S Lithium–Sulfur Batteries in Fresh and 500-Cycled

diffusion coefficients	fresh Super P/S **(10** ^**–8**^ cm^**2**^ **s** ^**–1**^ **)**	Super P/S after 500 cycles **(10** ^**–8**^ **cm** ^**2**^ **s** ^**–1**^ **)**	fresh AlPC_12_/S **(10** ^**–8**^ **cm** ^**2**^ **s** ^**–1**^ **)**	AlPC_12_/S after 500 cycles **(10** ^**–8**^ **cm** ^**2**^ **s** ^**–1**^ **)**
***D*_Li_^A^**	2.47	1.03	2.49	1.34
***D*_Li_^B^**	17.6	10.6	22.1	13.8
***D*_Li_^C^**	11.4	1.83	14.5	8.79

As shown in Table 2, the material AlPC_12_ offers several distinct advantages compared to other cathode materials from the literature. AlPC_12_ exhibits an initial capacity of 791.2 mAh/g and retains a final capacity of 530.6 mAh/g after 500 cycles, which is notably higher than the capacities of the other materials tested under the same C-rate conditions (0.5 C). For example, TiO_2_–TiN demonstrates a final capacity of 514.4 mAh/g,^60^ and Fe_2_O_3_/SnO_2_ ODs@S retains a capacity of 350 mAh/g after 500 cycles,^61^ both of which are lower than that of AlPC_12_. The C-rate employed in this comparison further underscores the superior performance of AlPC_12_. Despite being evaluated at a moderate C-rate of 0.5 C, which facilitates faster charge/discharge cycles, AlPC_12_ outperforms most of the listed materials in terms of capacity. Typically, a higher C-rate is associated with a reduction in capacity due to the increased cycling speed. However, AlPC_12_ maintains a significant capacity advantage even under these conditions. Moreover, AlPC_12_ demonstrates an exceptional decay rate of 0.06% per cycle, one of the lowest among the listed materials. This decay rate, combined with its stability over 500 cycles, underscores the material’s durability and long-term cycling stability. In contrast, several other materials, such as S/CO_3_O_4_-CoP-CNT^62^ and Co/CoV_2_O_4_/NC@S,^63^ exhibit higher decay rates and maintain capacity for only 100–200 cycles, further highlighting the robustness of AlPC_12_ for extended cycling applications.

**Table 2:** Comparative Performance of Various Cathode Materials for Lithium–Sulfur Batteries from Literature

cathode	initial capacity (mAh/g)	final capacity (mAh/g)	cycle number	C-rate	decay rate (%/cycle)	reference
AlPC_12_/S	791.2	530.6	500	0.5	0.06	this work
TiO_2_–TiN/S	774	514	500	0.5	0.07	^60^
A_5_HNT@NC/S	691.2	580.4	150	0.5	0.107	^61^
Fe_2_O_3_/SnO_2_ ODs@S	500	350	500	1	0.06	^64^
S/CoO-CoP-CNT	979.9	367.9	200	0.2	0.31	^62^
Co/CoV_2_O_6_/NC@S	558.4	395.1	100	0.2	0.29	^63^
VO_2_–VS_2_ @S	854	432	500	1	0.09	^65^
S/Co_9_S_8_-Mo_2_C	609.4	340.5	200	1	0.22	^66^
PNCS/NG-CI/S	875.5	507	500	1	0.08	^67^
BCS*T*/KB/S	904.8	603.4	200	1	0.17	^68^
Fe_2_P-NPC	800	495	200	0.1	0.24	^69^
1D-Fe3O4@C/S	791	627.6	200	0.5	0.115	^70^

## Conclusions

The AlPC_12_ composite was synthesized using a hydrolytic condensation method, notable for its simplicity and scalability, making it suitable for practical application. The synthesized AlPC_12_/S battery shows exceptional performance metrics, including a high discharge capacity and impressive cyclability, even with higher sulfur loading. DFT simulations indicate a strong interaction between AlPC_12_ and LiPS, which immobilizes the LiPS within the cathode and prevents their diffusion through the separator toward the anode. This interaction contributes to a lower capacity decay rate and enhances the overall electrochemical performance of the battery. This research underscores the significance of a 3D bulk structure within a composite for the effective management of LiPS, showing a direct approach to developing a robust LiPS adsorbent that acts as an efficient sulfur host. The methodologies and findings from this study are poised to make a substantial impact on the advancement of high-performance cathode materials for lithium–sulfur batteries. Given the growing demand for energy storage solutions that are both high-capacity and long-lasting, the development of such advanced materials is crucial. This study not only contributes to the scientific understanding of Li–S battery chemistry but also provides a practical pathway for the commercial viability of these batteries, supporting the transition to sustainable energy systems and facilitating the adoption of renewable energy sources and electric vehicles, ultimately addressing global energy challenges and contributing to a more sustainable and energy-efficient future.

## Supplementary Material







## Notes

Supplementary SEM images, BET isotherm, thermogravimetric analysis, XRD pattern, and supplementary electrochemical performance of different electrodes (PDF)
Visible battery test of AlPC_12_ (MP4)
Super P (MP4)
