# Supplementary material for: Suppressing Lithium Polysulfide Shuttle in Li–S Batteries Using the AlPC12 Composite for Enhanced Stability and Performance
Source: ACS Appl Mater Interfaces. 2025 May 21;17(22):32293–305. doi: 10.1021/acsami.5c02942 (PMC12147080; doi:10.1021/acsami.5c02942)
Supplement: Supplementary file 1 [file am5c02942_si_001.pdf]

Suppressing Lithium Polysulfide Shuttle in Li-S Batteries Using AlPC$_{12}$ Composite for Enhanced Stability and Performance

Supporting Information

Ka Chun LI[a], Yaoqi WEI[a], Xuanming CHEN[a], Zeyuan DI[a], Chi Ho WONG[b], Leung Yuk Frank Lam[a,*], Xijun HU[a,*]

[a,]Department of Chemical and Biological Engineering, The Hong Kong University of Science and Technology, Kowloon 999077, Hong Kong, China

[b,]Division of Science, Engineering and Health Studies, School of Professional Education and Executive Development, The Hong Kong Polytechnic University, Hong Kong, China

*Email address: kefrank@ust.hk; kexhu@ust.hk

Supporting Figures

The Li-S coin cells were disassembled using a hydraulic crimper after replacing the mold. All operations were carried out inside an argon-filled glovebox to prevent air exposure. After cycling, both the cathode and lithium anode were carefully removed from the cells and cleaned with dimethoxyethane (DME) to remove residual electrolytes. The central areas of the electrodes were cut and selected for further characterization. Scanning electron microscopy (SEM) was performed to analyze the surface morphology (**Figure S1**). Due to the high sensitivity of lithium metal to oxygen, the anode samples were transferred using an inert gas transfer chamber and handled exclusively in the argon-filled glovebox to prevent oxidation during sample preparation.

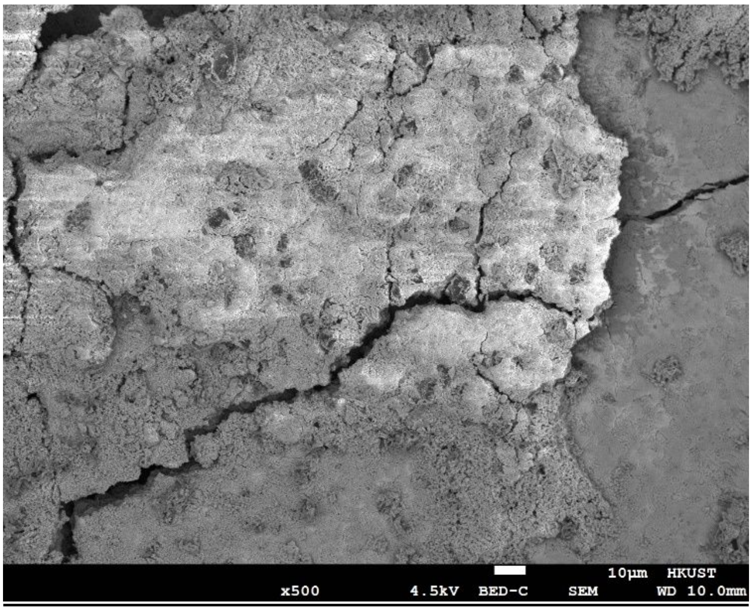

Figure S1. The SEM images of Li anodes of $AlPC_{12}/S$ battery after cycling.

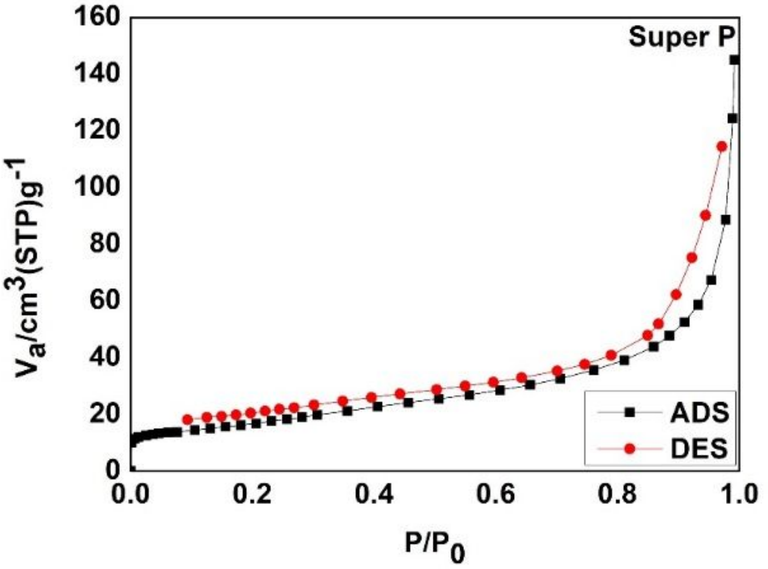

Figure S2. BET isotherm of Super P.

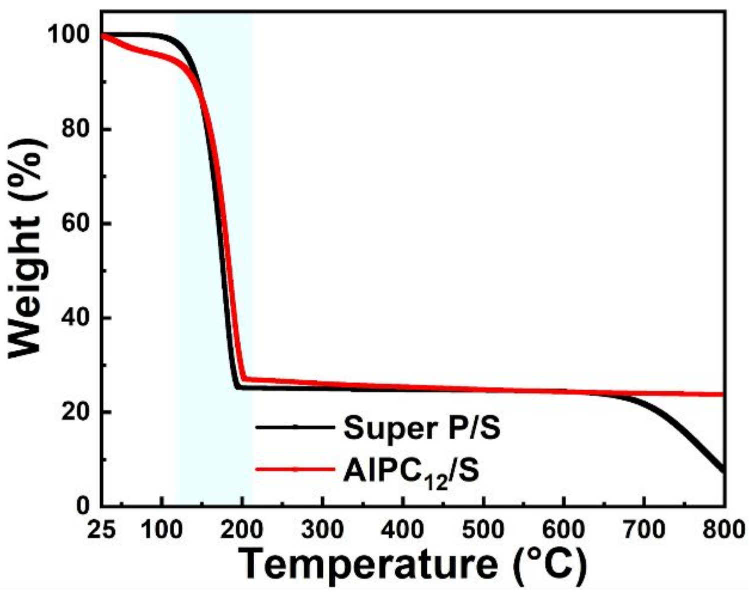

Figure S3. TGA curve of $AlPC_{12}/S$ composite and Super P/S composite.

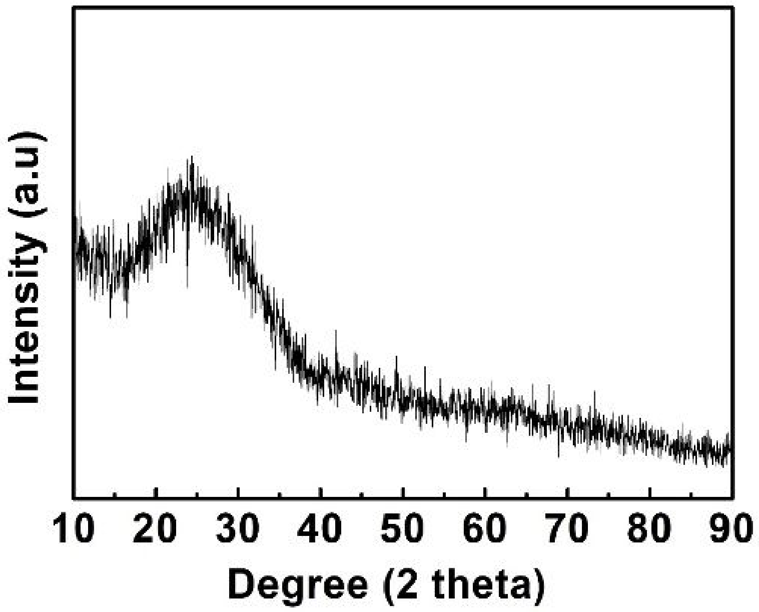

Figure S4. XRD pattern of $AlPC_{12}$ composite.

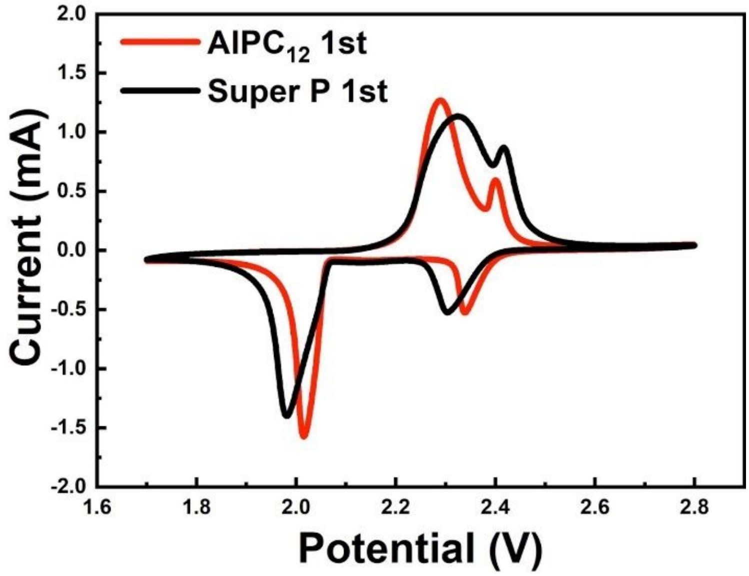

Figure S5. First-cycle charge-discharge profiles of $AlPC_{12}$/S and Super P/S batteries.

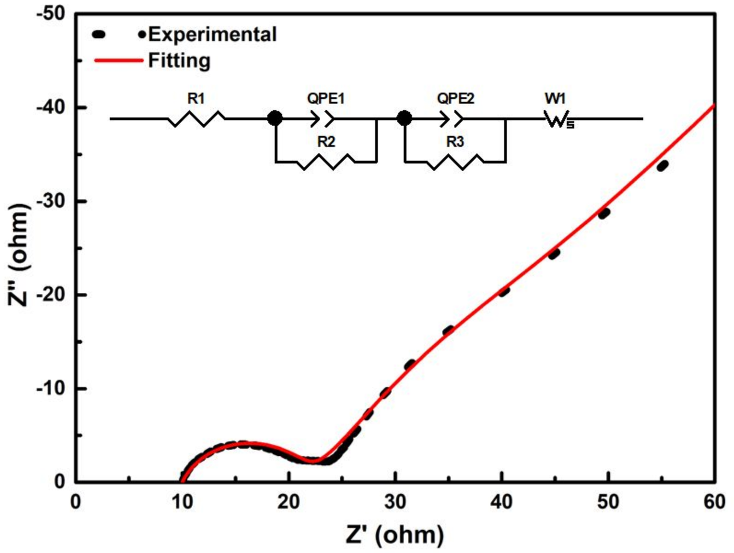

Figure S6. Nyquist plots of $AlPC_{12}/S$ battery after 500 cycles.

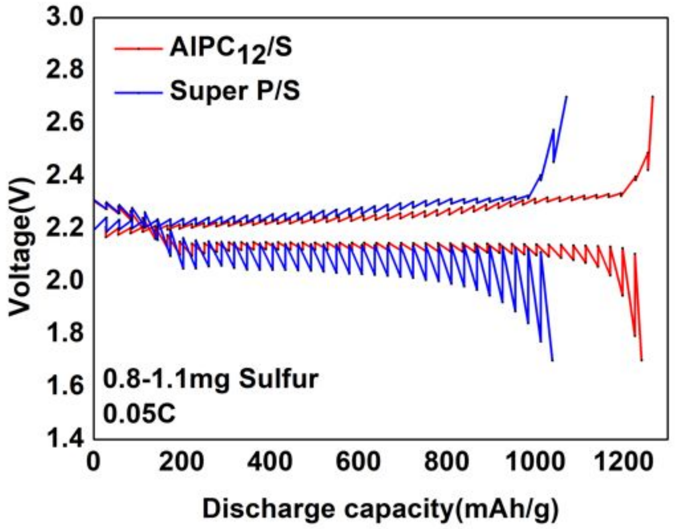

Figure S7. GITT performance of $AlPC_{12}/S$ battery and Super P/S battery.

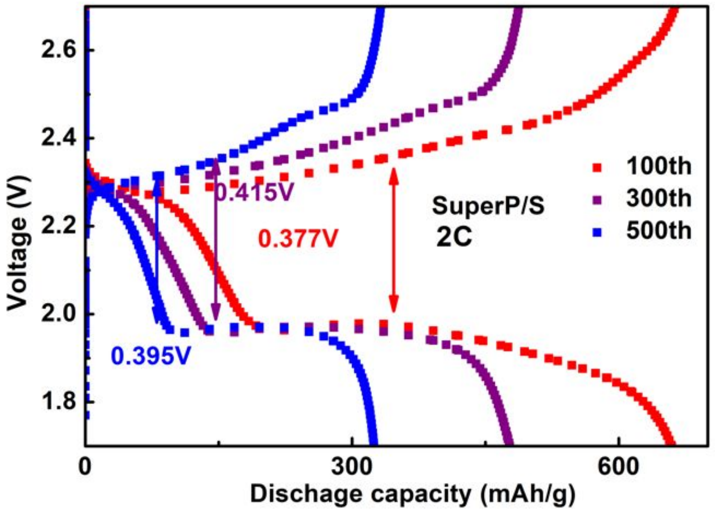

Figure S8. Galvanostatic charge and discharge profiles of SuperP/S battery at different cycle numbers in 2C.

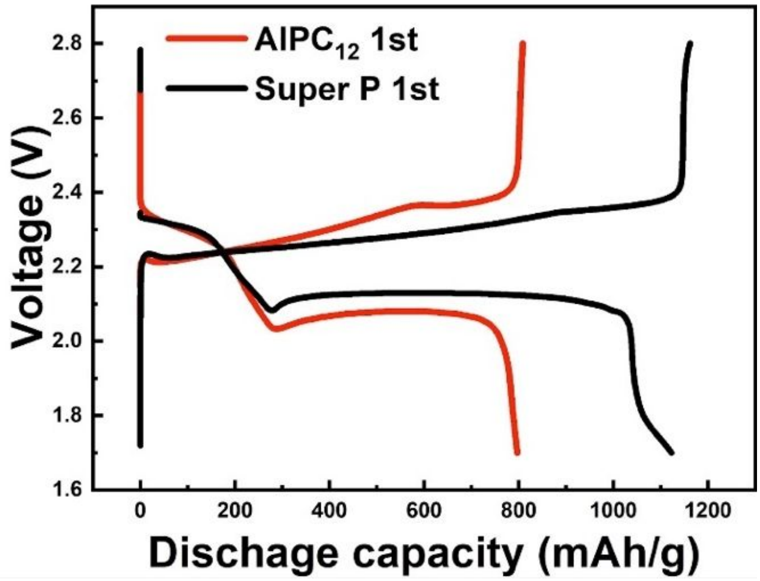

Figure S9. First-cycle CV curves of $AlPC_{12}$/S and Super-P/S batteries.

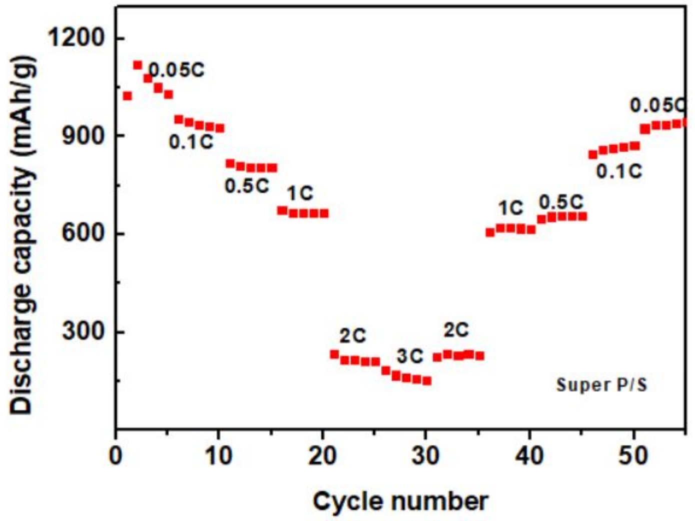

Figure S10. The rate performance of the Super P/S cathode.

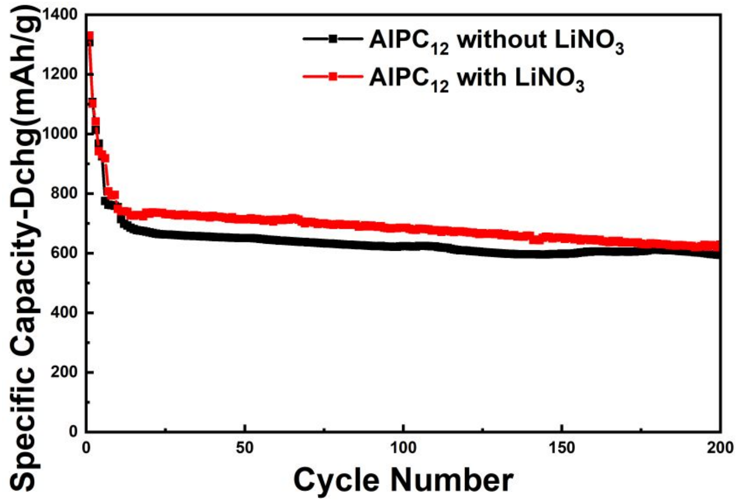

Figure S11. Cyclic performance test on $AlPC_{12}$/S battery with /without $LiNO_3$ added electrolyte.

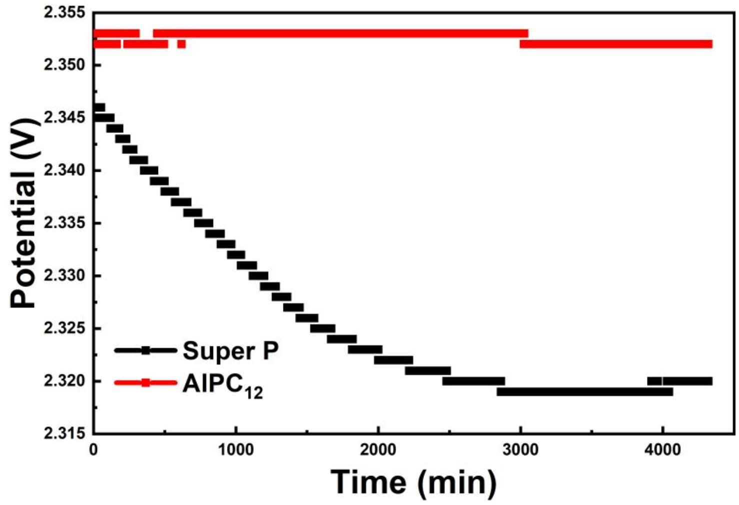

Figure S12. OCP self-discharge profile of $AlPC_{12}$/S and Super P/S batteries.
